# Femoral anteversion change is associated with ischiofemoral impingement after total hip arthroplasty: a retrospective CT evaluation

**DOI:** 10.1007/s00330-023-10428-2

**Published:** 2023-11-10

**Authors:** Adrian A. Marth, Sophia S. Goller, Reto Sutter

**Affiliations:** 1 Swiss Center for Musculoskeletal Imaging, Balgrist Campus AG, Zurich, Switzerland; 2 Department of Radiology, Balgrist University Hospital, Faculty of Medicine, University of Zurich, Zurich, Switzerland

**Keywords:** Arthroplasty (replacement, hip), Pain (postoperative), Tomography (X-ray computed)

## Abstract

**Objectives:**

We evaluated the relationship between femoral anteversion (FA), FA change, and ischiofemoral impingement (IFI) and the relationship between FA, femoral offset (FO), and greater trochanteric pain syndrome (GTPS) after total hip arthroplasty (THA).

**Materials and methods:**

In this retrospective study, two readers assessed FA and FO on CT images of 197 patients following primary THA with an anterior surgical approach between 2014 and 2021. FA change was calculated relative to preoperative CT, while FO change was calculated relative to preoperative radiographs and classified as decreased (≥−5 mm), increased (≥ + 5 mm), or restored (± 5 mm). Clinical and imaging data were analyzed for IFI and GTPS after surgery. Group differences were evaluated using Student’s *t*-test, chi-square analysis, and receiver operating characteristic (ROC) analysis.

**Results:**

The change in FA was 3.6 ± 3.3° to a postoperative FA of 22.5 ± 6.8°, while FO increased by 1.7 ± 3.5 mm to a postoperative FO of 42.9 ± 7.1 mm. FA and FA change were higher in patients with IFI (*p *≤ 0.006), while no significant difference was observed for patients with and without GTPS (*p *≥ 0.122). IFI was more common in females (*p *= 0.023). In the ROC analysis, an AUC of 0.859 was observed for FA change to predict IFI, whereas the AUC value was 0.726 for FA alone. No significant difference was found for FO change in patients with and without IFI or GTPS (*p* ≥ 0.187).

**Conclusion:**

Postoperative FA, FA change, and female sex were associated with IFI after anterior-approached THA. The change in FA was a better predictor of IFI than absolute postoperative FA alone.

**Clinical relevance statement:**

The findings of this study suggest that preservation of the preoperative femoral anteversion may reduce postoperative ischiofemoral impingement in patients undergoing total hip arthroplasty.

**Key Points:**

*• Higher postoperative femoral anteversion and anteversion change were associated with ischiofemoral impingement.*

*• Femoral anteversion change was a better predictor of impingement than absolute postoperative anteversion.*

*• No significant association was found between femoral offset and postoperative hip pain.*

## Introduction

The number of total hip arthroplasty (THA) procedures has increased worldwide due to the rising life expectancy, making it one of the most commonly performed orthopedic procedures [1]. Most patients experience good pain relief and restoration of hip function after surgery. Nevertheless, a subset of patients experience persistent or even newly emerging hip pain after surgery.

Hip pain after THA can be caused by various factors such as aseptic loosening, instability, infection, periprosthetic fracture, or penetrating acetabular screws [2]. Lateral hip pain occurs frequently after THA and is denoted as greater trochanteric pain syndrome (GTPS) in the current literature [3]. GTPS is experienced by approximately 4–17% of patients following THA [3, 4] and is believed to result either from iatrogenic damage to the tendons of the hip abductors and short external rotators or from degenerative abductor tendon changes potentially affecting the subgluteal bursae, resulting in an inflammatory reaction [5]. Ischiofemoral impingement (IFI) manifests as buttock and groin pain [6] and originates from a soft-tissue compression of the quadratus femoris muscle between the lesser trochanter and the ischial tuberosity [7–9].

Although surgical and demographic factors, such as surgical approach [10], cup overhang [11, 12], and female sex [8, 13], have been recognized as possible causes of GTPS after THA, the role of the femoral offset change (FO) has been investigated in only a limited number of studies, and these studies have produced conflicting results [14–17]. On the other hand, higher femoral anteversion (FA) after THA is suspected to be associated with IFI [18], but it is not known whether this association is also present with the now common anterior surgical approach. Furthermore, the effect of changing FA on hip pain following THA has not yet been reported. In light of this data gap, the purpose of this study was to evaluate the impact of FA and FO change as measured on CT images on the occurrence of IFI and GTPS in patients after undergoing anterior-approached THA.

## Materials and methods

### Patient selection

This retrospective study was approved by the local ethics committee (Cantonal Ethics Committee Zurich) and was conducted in accordance with national ethical guidelines and the Helsinki Declaration of 1964 and its subsequent amendments.

**Fig. 1:**
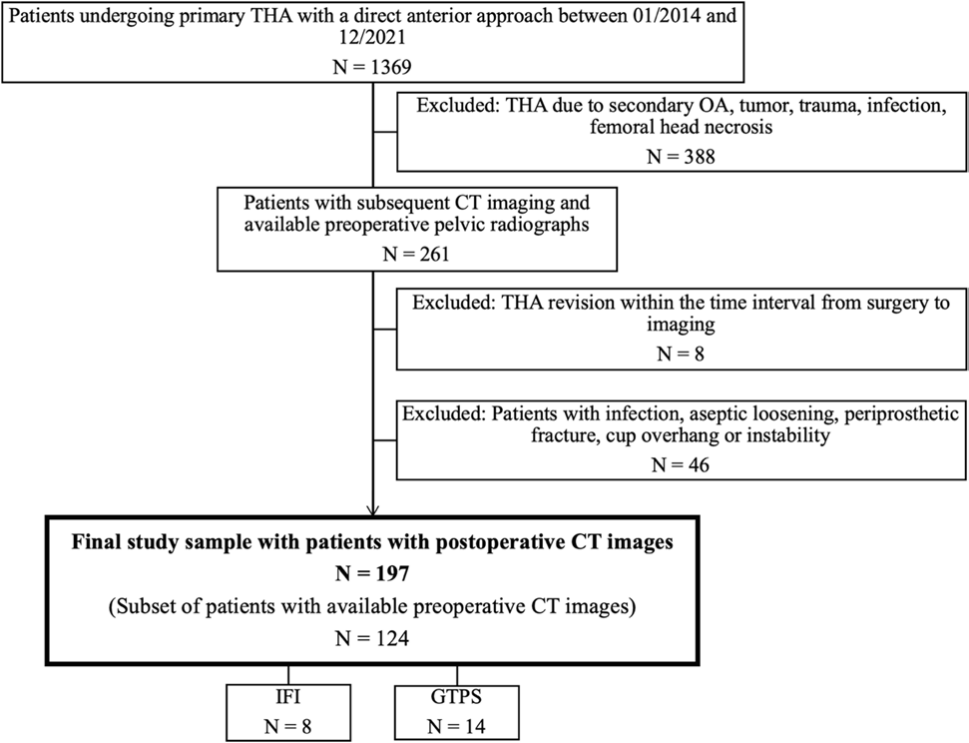
Flowchart depicting the patient selection process. IFI, ischiofemoral impingement; GTPS, greater trochanteric pain syndrome; OA, osteoarthritis; THA, total hip arthroplasty

Figure 1 illustrates the entire patient selection process. To identify patients for the present study, the hospital information system was queried to retrieve records of all patients who underwent THA between 2014 and 2021. Subsequently, only patients who received THA through an anterior approach were included, while patients who underwent THA due to secondary osteoarthritis (e.g., hip dysplasia), tumor, trauma, infection, or femoral head necrosis or had a history of THA revision were excluded from this study. Patients who had received pre-surgical pelvic radiographs and post-surgical CT examinations of the pelvis and knee region were selected from the local Picture Archiving and Communications System (Merlin, Phoenix PACS). A total of 261 patients were identified for further analysis based on these criteria. CT was performed for complications after THA, for postoperative assessment of positioning of THA components, or for other clinical indications. Out of this sample, patients with THA revision, as well as documented infection, aseptic loosening, periprosthetic fracture, cup overhang, or instability as a reason for groin pain, were excluded, leading to a sample size of 197 patients. Of these patients, 124 had preoperative CT images available to assess FA change. Moreover, a review of surgical and clinical reports as well as imaging data (magnetic resonance imaging and ultrasound) of the final patient cohort was conducted, aiming to identify documented cases of IFI and GTPS. Clinical diagnoses were made exclusively by orthopedic surgeons at the local institution specializing in hip surgery. For IFI, the diagnosis was made based on the patients’ history, symptoms, and specific clinical examination (long-stride walking test and/or IFI test) [19, 20]. For GTPS, all clinical diagnoses were made by an orthopedic surgeon specialized on hip surgery. The diagnosis was based on the patient’s symptoms (location of pain and/or tenderness, worsening of pain with unilateral weight-bearing activities, lying on the affected side) and clinical examination (hip abduction and external rotation test, FABER test, resisted external rotation test, or Trendelenburg test), in accordance with the 2022 ISHA Physical Therapy Agreement for the Evaluation and Management of GTPS [21]. IFI and GTPS were considered confirmed only if a clinical or surgical report documented these conditions and the corresponding imaging showed signs corroborating the suspected clinical diagnosis. This was edema and/or fatty atrophy of the quadratus femoris muscle for IFI [8] respectively abductor tendinopathy and/or trochanteric bursitis for GTPS [22, 23] in postoperative ultrasound or MR imaging studies. In addition, imaging studies of patients without symptomatic IFI or GTPS in this population were reviewed for radiologic signs of these diagnoses. For IFI, this was presence of fatty atrophy of the quadratus femoris muscle and measurement of the ischiofemoral space on the surgically treated side according to Torriani et al [8] on postoperative CT images. The measured values were then compared to normative values for the ischiofemoral space established by Özdemir et al [24]. For GTPS, available postoperative ultrasound and MRI examinations were reviewed for signs of trochanteric bursitis and abductor tendinopathy as described above [22, 23].

### Image analysis

CT scans were acquired on 64-slice and 128-slice CT scanners (Somatom Edge Plus, Philips Brilliance 64, Somatom Definition AS Plus) at 120–140 kVp with and without contrast agent for different clinical indications, referred by in-house or external orthopedic surgeons or external partner hospitals. Two fellowship-trained radiologists evaluated all CT images and performed measurements within the local Picture Archiving and Communications System viewer.

**Fig. 2:**
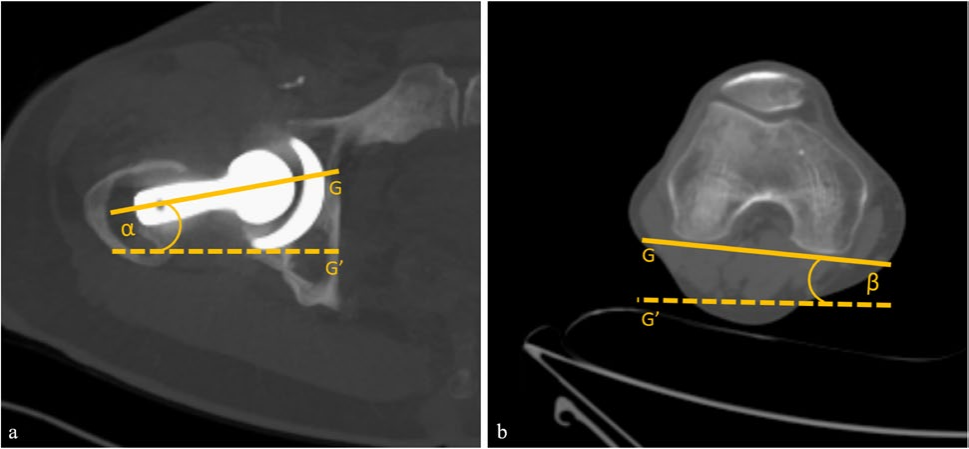
Calculation of femoral anteversion on axial CT images. The angle was defined by lines that were drawn through the prosthesis neck and the center of the prosthesis head (**a**) as well as through the posterior tangent connecting the most distal points of the femoral condyles (**b**). For calculating the femoral anteversion, two angle measurements were taken using a horizontal line (*G*′) as a fixed reference for the femoral neck and femoral condyle axis. The resulting angles were then added or subtracted, depending on whether they were oriented in the same or opposite direction

FA measurement was conducted with the following approach (Fig. 2): First, the prosthesis head center, prosthesis neck, and the most distal portion of the femoral condyles were identified as reference points on the CT images. The femoral anteversion was measured according to Murphy et al as the angle between lines that were drawn through the center of the base of the femoral neck and the center of the prosthesis head aided by maximal intensity projection as well as through the posterior tangent connecting the most distal points of the femoral condyles (“tabletop method”) [25]. The same method was used for pre-surgical CT images. Subsequently, the absolute difference between the pre-and post-surgical images was computed.

**Fig. 3:**
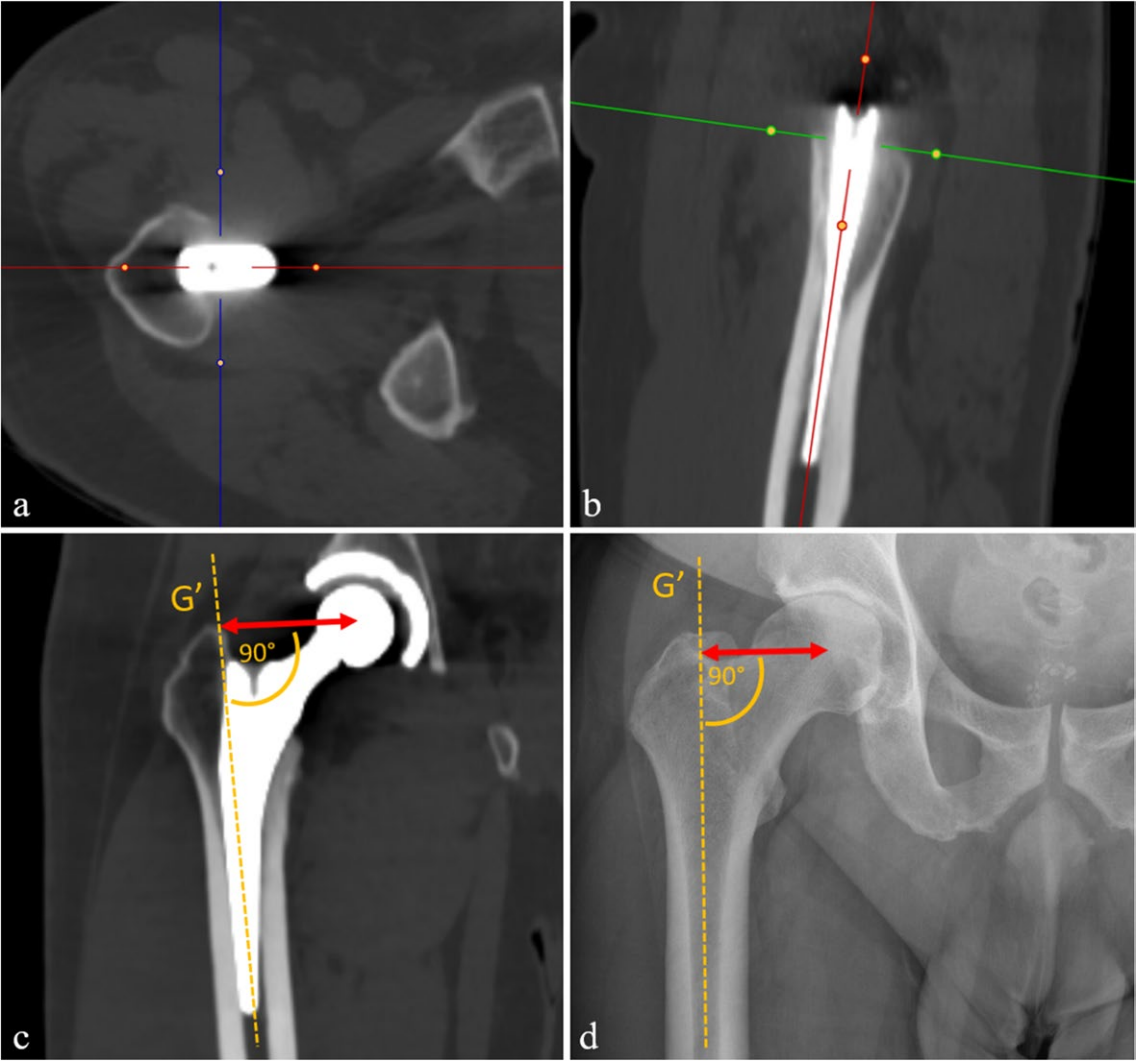
For calculation of femoral offset on CT images, these were adjusted utilizing multiplanar reformation along the axes of the prosthesis shaft in the axial and sagittal plane (**a**, **b**). Subsequently, a line was drawn along the femoral shaft (*G*′), intersecting the most prominent point of the greater trochanter. Femoral offset (double-arrow) was then calculated as the distance between the femoral head center of rotation and a line bisecting the femoral long axis (**c**). The same method for calculation of the femoral offset (double-arrow) was applied for pre-surgical radiographs of the pelvis (**d**)

For the calculation of FO, CT images were adjusted utilizing multiplanar reformation along the axes of the prosthesis shaft in the axial and sagittal plane (Fig. 3). FO was then calculated as the distance between the center of rotation of the femoral head and a line bisecting the long axis of the femur [26]. For anteroposterior radiographs of the pelvis obtained before surgery, the same measurement method was conducted and the FO difference between pre-and post-surgical images was calculated. A magnification marker was available for all radiographs to prevent measurement inconsistencies. Patients were categorized into three groups according to the current literature [14, 15]: (1) patients with an FO decrease greater than 5 mm, (2) patients with a restored FO between −5 mm and +5 mm, and (3) patients with an FO increase greater than 5 mm.

### Statistical analysis

SPSS Statistics (v25, IBM Corporation) was utilized for all statistical analyses. To test for normal distribution of continuous variables, the Shapiro-Wilk test was employed. If normally distributed, variables are presented as mean ± standard deviation, whereas non-normally distributed are presented as median with interquartile range in parentheses. For group comparisons, Student’s *t*-test was used for normally distributed variables, while categorical variables were assessed using chi-square analysis. For ischiofemoral space measurement, a one-tailed *t*-test was employed for comparison with the normative value. In cases of significance, the predictive performance of femoral measurements was evaluated using receiver operating characteristic curves. The area under the curve (AUC) was used as a measure of accuracy, with AUC values greater than 0.8 considered “excellent,” values between 0.7 and 0.8 considered “fair,” and values less than 0.7 considered “poor” [27]. At an alpha level of ≤ 0.05, the observed results were considered statistically significant. Interreader agreement was assessed with a kappa statistic (Fleiss’ Kappa) and the level of agreement was reported according to Landis et al [28].

## Results

### Patient characteristics

**Fig. 4:**
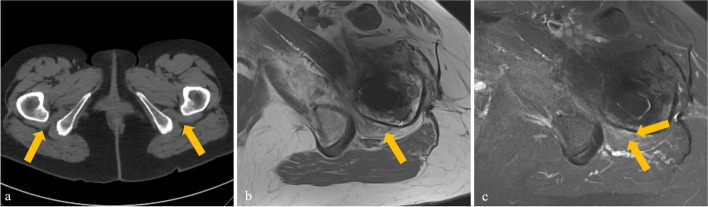
A 72-year-old female patient admitted for buttock pain following THA with clinical suspicion of IFI. Preoperative CT imaging depicting no relevant narrowing of the left ischiofemoral space compared to the contralateral side (arrows) or fatty atrophy of the quadratus femoris muscle (**a**). Postoperative axial T1-weighted MR image shows fatty atrophy of the quadratus femoris muscle (arrow) and narrowing of the ischiofemoral space (**b**). The corresponding axial STIR image reveals an edema of the quadratus femoris muscle (**c**, arrows). IFI, ischiofemoral impingement; STIR, short-tau inversion recovery; THA, total hip arthroplasty

**Fig. 5:**
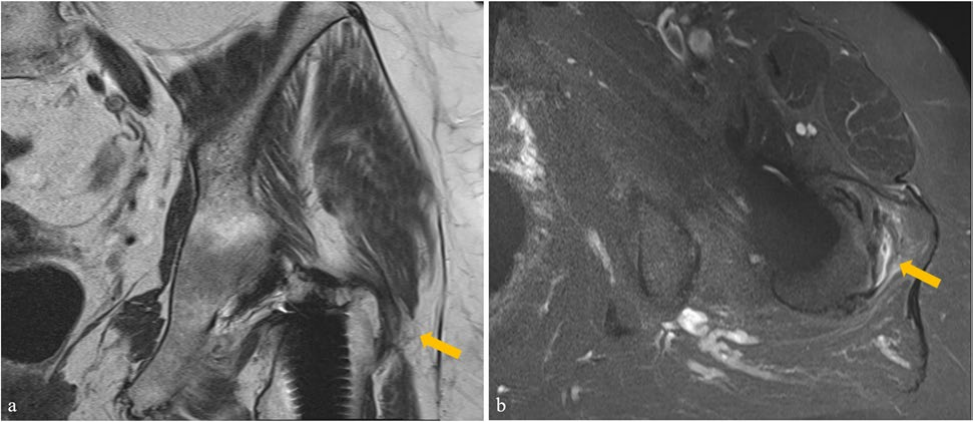
A 69-year-old female patient admitted for lateral hip pain following THA with clinical suspicion of GTPS. Coronal T1-weighted image (**a**) depicting partial detachment of the gluteus medius tendon at its insertion (arrow). Axial STIR image (**b**) reveals a fluid-filled trochanteric bursa with perifocal soft-tissue edema (arrow). GTPS, greater trochanteric pain syndrome; STIR, short-tau inversion recovery; THA, total hip arthroplasty

All demographic and clinical data are summarized in Table 1. The mean age at surgery was 62.4 ± 12.5 years and the mean time from surgery to CT imaging was 410 ± 646 days. GTPS was diagnosed in 14 patients (7.1%), while IFI was diagnosed in 8 patients (4.1%) (Figs. 4 and 5). There were no significant differences observed regarding age, sex, Body Mass Index and time from surgery to CT imaging between patients with and without IFI and GTPS (all *p* ≥ 0.366). Female sex was significantly associated with IFI (*p* = 0.006, odds ratio 8.034, 95% confidence interval (CI) 0.979–66.579), but not with GTPS (*p* = 0.488, odds ratio 1.471, 95% CI 0.491–4.409). CT examinations of individuals without symptomatic GTPS/IFI (*n* = 175) revealed a preoperative ischiofemoral space of 23.2 (5.6) mm, which did not differ significantly from the normative value previously described (*p* = 0.487) [24]. Fatty atrophy of the quadratus femoris muscle was noted in 7 cases (4.0%). For GTPS, ultrasound or MRI examinations of 33 patients without symptomatic GTPS/IFI (18.9%) were available for assessment. Of those, four patients (12.1%) exhibited signs of trochanteric bursitis and two patients (6.1%) exhibited gluteal tendinopathy.

**Table 1:** Demographic and clinical data as well as femoral anteversion and femoral offset obtained from CT images. Data are given as mean and standard deviation

Total study group (*n* = 197)	
Age (years)	62.4 ± 12.5
Female sex	95 (48.2)
Body mass index	28.3 ± 5.9
Time from surgery to CT imaging (days)	410 ± 646
Ischiofemoral impingement	8 (4.1)
Greater trochanteric pain syndrome	14 (7.1)
Femoral anteversion (°)	22.5 (6.8)
Femoral offset (mm)	
*Before surgery*	41.3 (6.5)
*After surgery*	42.9 (7.1)
*Offset increase*	1.7 (3.5)
Patients with pre- and postoperative CT images (*n* = 124)	
Preoperative femoral anteversion (°)	18.2 (4.4)
Femoral anteversion change (°)	3.6 (3.3)

Ordinal data is given as frequency with percentage in parentheses. Continuous data are presented as mean ± standard deviation as well as median with range or interquartile range in parentheses

### Femoral anteversion

**Fig. 6:**
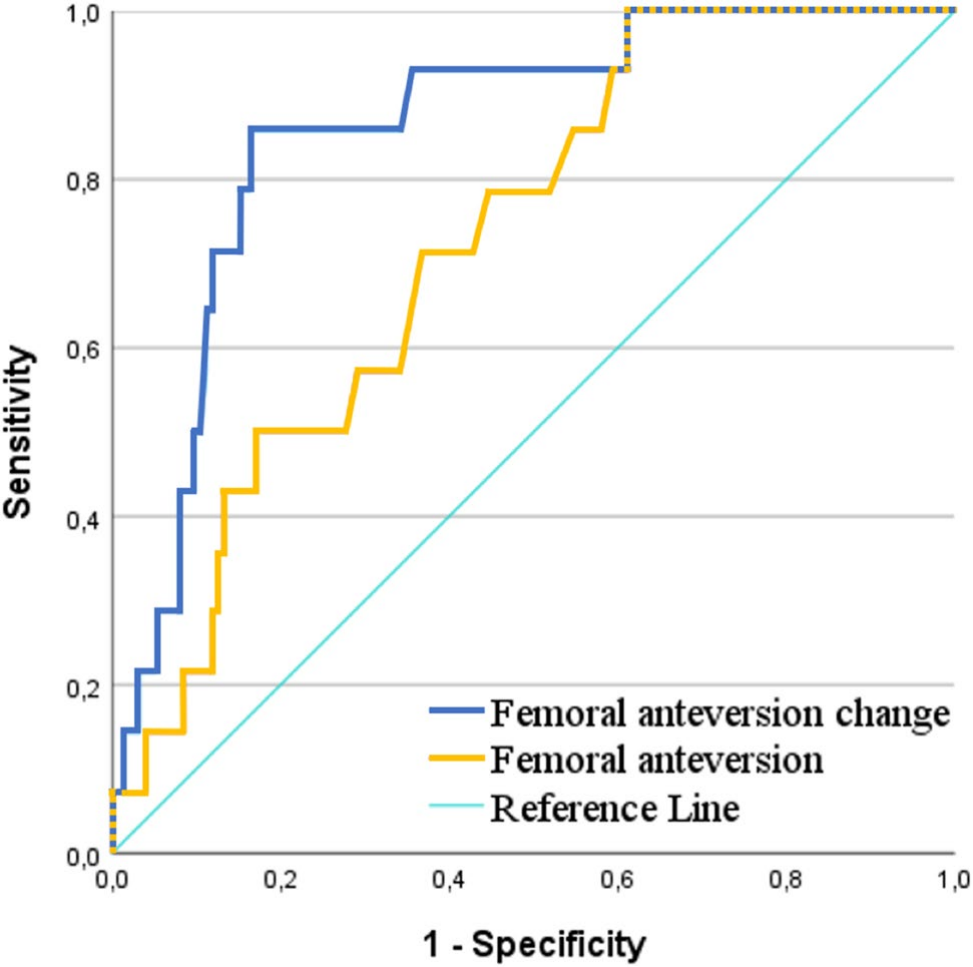
Receiver operating characteristic curve for postoperative femoral anteversion and femoral anteversion change. Predictive accuracy for the presence of IFI was excellent for femoral anteversion change (area under the curve (AUC) = 0.859, 95% CI 0.770–0.948) and moderate for absolute postoperative femoral anteversion (AUC = 0.726, 95% CI 0.611–0.842)

Mean FA was 18.2° (4.4°) before surgery and 22.5° (6.8°) after surgery, with a change of 3.6° (3.3°). Student’s *t*-test revealed that patients in the IFI group had higher postoperative FA angles (25.2° [5.9°]) compared to patients without IFI (14.4° [10.2°], *p* = 0.006, Table 2). FA change was 5.4° (3.2°) in the IFI group and 1.4° (3.1°) in the non-IFI group (*p* < 0.001). Differences in FA and FA change between patients with and without GTPS were non-significant (*p* ≥ 0.549). The receiver operating characteristic analysis revealed an excellent predictive power for FA change (AUC = 0.859, 95% CI 0.770–0.948) for identifying patients with IFI, whereas the predictive power for absolute FA values was moderate (AUC = 0.726, 95% CI 0.611–0.842) (see Fig. 6). Interreader agreement for both preoperative and postoperative CT images was almost perfect (κ = 0.813 and κ = 0.836, respectively).

**Table 2:** Group differences between patients with and without ischiofemoral impingement as well as greater trochanteric pain syndrome. Preoperative CT imaging to assess femoral anteversion change was available for all symptomatic patients

			*p* value	Odds ratio (95% CI)
	IFI (*n* = 8)	No IFI (*n * = 189)		
Age (years)	58.5 ± 11.5	62.6 ± 12.5	0.366	
Female sex	7 (87.5)	88 (46.6)	0.023*	8.034 (0.969–66.579)
Body mass index	28.3 ± 7.0	28.3 ± 5.8	0.994	
Time from surgery to CT imaging (days)	401 ± 635	421 ± 648	0.202	
Femoral anteversion (°)	25.2 (5.9)	14.4 (10.2)	0.006*	
Femoral anteversion change (°)	5.4 (3.2)	1.4 (3.1)	< 0.001*	
Offset change (mm)	+1.4 ([−2.8] – [+3.4])	+0.3 ([−0.4] – [+0.8])	0.419	
	GTPS (*n* = 14)	No GTPS (*n* = 183)		
Age (years)	64.3 ± 9.0	62.3 ± 12.7	0.562	1.471 (0.491–4.409)
Female sex	8 (57.1)	96 (52.5)	0.488	
Body mass index	25.8 ± 9.2	28.5 ± 5.5	0.105	
Time from surgery to CT imaging (days)	430 ± 639	398 ± 651	0.095	
Femoral anteversion (°)	13.5 (7.5)	15.3 (10.2)	0.549	
Femoral anteversion change (°)	3.0 (3.2)	2.4 (2.7)	0.751	
Offset change (mm)	+2.7 ([−0.8] – [+4.1])	+1.2 ([−1.1] – [+3.1])	0.059	

^*^Statistical significance

Ordinal data is given as frequency with percentage in parentheses. Continuous data are presented as mean ± standard deviation as well as median with range or interquartile range in parentheses. IFI, ischiofemoral impingement; GTPS, greater trochanteric pain syndrome

### Femoral offset

FO was 41.3 (6.5) mm before surgery and 42.9 (7.1) mm after surgery resulting in an increase of 1.7 (3.5) mm. Student’s *t*-test revealed no significant difference in absolute FO change between patients with and without pain (all *p* ≥ 0.059, Table 2). One hundred nineteen patients (60.7%) had a restored FO (± 5 mm), while the FO decreased ≥ 5 mm in 31 patients (15.7%) and increased ≥ 5 mm in 47 patients (23.9%, Table 3). Chi-square analysis revealed no difference between the different offset groups for IFI and GTPS, respectively (all *p* ≥ 0.187). Interreader agreement was substantial for both radiographs (κ = 0.725) and post-surgical CT images (κ = 0.782).

**Table 3:** Patient stratification according to femoral offset decrease (≥−5 mm), restoration (± 5 mm) or increase (≥ +5 mm) on CT images after surgery compared to pre-surgical radiographs

Offset			*p* value	Odds ratio (95% CI)
	IFI (*n* = 8)	No IFI (*n*= 189)		
Decreased	1 (12.5)	29 (15.4)	0.759	0.457 [0.055–3.816]
Restored	4 (50.0)	117 (61.9)	0.459	0.386 [0.090–1.664]
Increased	3 (37.5)	45 (23.8)	0.187	0.551 [0.087–2.431]
	GTPS (*n* = 14)	No GTPS (*n* = 183)		
Decreased	2 (14.2)	30 (16.4)	0.837	0.850 [0.181–3.994]
Restored	8 (57.1)	110 (60.1)	0.827	0.885 [0.295–2.656]
Increased	4 (28.7)	42 (23.0)	0.632	1.343 [0.401–4.502]

Data are presented as frequency with percentage in parentheses. IFI, ischiofemoral impingement; GTPS, greater trochanteric pain syndrome

## Discussion

The first major finding of this study was that IFI occurred significantly more often in THA patients with a higher postoperative FA, and especially with a larger FA change. The main etiology of pain in patients with IFI is attributed to the mechanical compression of the quadratus femoris muscle by narrowing of the ischiofemoral space, which is caused by various factors, including congenital conditions (e.g., coxa valga, developmental dysplasia of the hip), acquired deformities (e.g., severe osteoarthritis), and iatrogenic causes such as THA [29, 30]. In THA, quadratus femoris entrapment due to a decrease of the ischiofemoral space can be severe, as the postoperative anteversion angles of the femur exhibit a significant variability [31]. Additionally, biomechanical factors such as abductor insufficiency (e.g., due to degenerative or iatrogenic partial tear) can contribute to a “dynamic” mechanical conflict as a consequence of increased hip adduction [32, 33]. These biomechanical considerations offer a plausible explanation for the association between higher FA and IFI. However, the current understanding of the influence of FA change following THA is limited. This knowledge gap becomes particularly relevant when considering the variability of postoperative FA, which is influenced by factors such as stem design and cementation, with the greatest variability observed in patients with cementless THA as in the present study [31]. It is worth noting that a rotational adjustment of the stem during surgery is better achieved with cemented stems because the liquid cement allows for a degree of rotation both during and after the complete placement of the stem. In everyday clinical practice, the surgeon often positions relative to the condylar plane of the femur by eye adhering to a “safe zone” as described by Lewinnek et al [34]. However, the use of mechanical insertion devices, intraoperative fluoroscopy, or imageless navigation has been advocated for a more precise component placement [35]. The high predictive power of FA change observed in this study therefore underpins the importance of a correct stem placement along with the use of preoperative CT imaging for THA, suggesting that restoring preoperative FA levels may aid in minimizing the occurrence of postoperative IFI.

In our study, the occurrence of IFI was less frequent (4.1%) compared to the incidence in other studies (ranging from 6 to 7.2%) [18, 36]. This phenomenon might be attributable to the choice of surgical approach: The anterior approach is located between the tensor fascia latae and the sartorius muscle and therefore spares the abductor muscles during surgery. As a result, a lower frequency of abductor tendon detachments, abductor partial tears, and tendinopathy of the gluteus medius and minimus muscles are present when compared to other surgical approaches [37]. Therefore, this might lead to a lower risk of the previously mentioned “dynamic” mechanical conflict, especially because the minimum ischiofemoral space is smaller during dynamic activities than at rest [33, 38]. The observed sex-specific difference with an OR of 8.034 for females to develop IFI is in line with the current literature [7, 8]. This is mainly believed to be due to sex-specific pelvic anatomic variations such as a wider intertuberal distance and lesser neck-shaft angle in females, which are associated with a reduced ischiofemoral distance [24].

The second major finding of our study was that FO change following THA was not associated with GTPS. It is known that GTPS is associated with greater trochanteric bursitis, mostly resulting from an underlying abductor tendinopathy [39], but to date, there are diverging results regarding an association of GTPS to FO [14–17]. We hypothesized that an increased FO after THA explained the occurrence of GTPS, as it has been reported that friction between the greater trochanter and the overlying iliotibial band can lead to lateral hip pain [40]. However, our data confirm that a single measurement parameter such as FO is not sufficient to adequately identify symptomatic patients, as GTPS is also known to be influenced by other factors such as leg length discrepancy after surgery [41].

Even though this was not the primary endpoint of this study, we hypothesize that the choice of surgical approach plays a significant role in the development of GTPS, similar to patients with ischiofemoral impingement [10]. In particular, the absence of a need for incision through the fascia lata or the surgical release of the external rotators on the greater trochanter in anterior-approached THA might result in reduced scarring of the peritrochanteric soft tissues [41], thus leading to less pain, as a study by Iorio et al suggests [3].

Our study has the following limitations: First, even though we included a large cohort of 197 patients with postoperative CT examinations, postoperative IFI or GTPS was comparatively rare and only present in 22 patients. Second, the majority of postoperative CTs were performed due to complications after hip pain. However, we have made an effort to exclude all patients with identifiable causes of hip pain and only included patients in whom the clinical and radiological diagnosis of IFI and GTPS were concordant. Finally, establishing a correlation between the clinical identification of IFI and GTPS and their radiologic diagnosis remains challenging, as even asymptomatic individuals may exhibit signs of these conditions without experiencing symptoms.

## Conclusion

Our study examined the impact of FA and FO on IFI and GTPS following anterior-approached THA. We observed an association between higher FA, higher FA change, female sex, and the occurrence of IFI. The comparatively low overall incidence of IFI in our study might be attributed to the muscle-sparing surgical approach that avoids detachment of abductor tendon insertions. FO change after surgery did not show an association with GTPS, suggesting that this condition might rather be influenced by multiple biomechanical and surgical factors following THA and cannot be attributed to one measurement parameter solely.

## Abbreviations

AUC: Area under the curve
CI: Confidence interval
FA: Femoral anteversion
FO: Femoral offset
GTPS: Greater trochanteric pain syndrome
IFI: Ischiofemoral impingement
THA: Total hip arthroplasty

## Declarations

### Guarantor

The scientific guarantor of this publication is Reto Sutter, MD.

### Statistics and biometry

No complex statistical methods were necessary for this paper.

### Study subjects or cohorts overlap

No study subjects or cohorts have been previously reported.

### Methodology

• retrospective

• observational

• performed at one institution
